# Grey Wolf (*Canis lupus*) Recolonization in Hungary: Does the Predation Risk Affect the Red Deer (*Cervus elaphus*) Population?

**DOI:** 10.3390/ani14243557

**Published:** 2024-12-10

**Authors:** Zsolt Biró, Krisztián Katona, László Szabó, Dávid Sütő, Miklós Heltai

**Affiliations:** 1 Institute for Wildlife Management and Nature Conservation, Hungarian University of Agriculture and Life Sciences, Páter Károly utca 1, H-2100 Gödöllő, Hungary; katona.krisztian@uni-mate.hu (K.K.); szabo.laszlo@uni-mate.hu (L.S.); heltai.miklos.gabor@uni-mate.hu (M.H.); 2 WWF Hungary, Álmos Vezér útja 69/A, H-1141 Budapest, Hungary; david.suto@wwf.hu

**Keywords:** large carnivore, stress hormone, kidney fat index, bone marrow

## Abstract

The populations and distribution areas of large carnivores have declined all over the world due to extirpation and habitat alteration and degradation. However, the grey wolf (*Canis lupus*) has recovered in Europe in recent decades and has been reappearing in Hungary since the 1990s. Since the dominant prey of this carnivore is the red deer (*Cervus elaphus*) and the wild boar (*Sus scrofa*) in Central and Eastern Europe, we aimed to study the impact of wolves on local deer populations. Based on hunters’ opinions, we expected an increasing wolf presence and intense effects of wolves on the stress level and body condition of deer. First, we examined the occupied area by wolf in the North Hungarian Mountains. The distribution map was based on a questionnaire among the game managers. To measure the influence of the reappearing predator population on the red deer individuals, we estimated the body condition (kidney fat and bone marrow index) and stress hormone level of faecal samples. We compared them between the areas colonised by wolves and control sites in the mountains. We revealed an increased distribution area of wolves in the mountains since 2014. The stress hormone level was lower in the wolf-free sites in the case of faeces gathered from the ground, but there was similar amount of cortisol in the samples collected from the hunted animals. The body condition indices were not different between the two area types (average kidney fat index > 100% and almost 100% for the bone marrow fat content). Our results do not support a very intense recent impact of the wolf population on the body condition and stress level of red deer in Hungary.

## Simple Summary

The range and population size of the grey wolf has increased in recent decades in Europe, and the species has also reappeared in Hungary. According to hunters, the increasing presence of wolves in the North Hungarian Mountains has a significant impact on the deer population. We investigated how stressed the red deer are by the wolves and whether their condition is worse in wolf areas than in sites where the predator is not present. The questionnaire among the hunters revealed the increase in the range of the wolves. The faecal samples of the deer collected from the ground in the area where wolves were not present showed lower stress levels than in other areas. The body fat reserves of the deer individuals were equal in wolf areas and wolf-free sites. Our results do not support a very intense recent impact of the wolf population on the body condition and stress level of red deer in Hungary.

## 1. Introduction

The populations and distribution areas of large carnivores have declined all over the world due to extirpation and habitat degradation and fragmentation [1,2]. However, the habitat restorations and the conservation actions (establishing protected areas, mitigations of human–carnivore conflicts) have resulted in recovery for the grey wolves (*Canis lupus*) in Europe and also in North America [2,3,4,5]. Increasing forested areas which provide more cover and the abandonment of rural areas due to urbanisation [6,7] caused an increase in the ungulate prey populations like red deer (*Cervus elaphus*) or roe deer (*Capreolus capreolus*) [8]. Moreover, these environmental changes increased the number of possible denning sites and shelters for wolves and decreased the human persecution [7].

A suitable habitat and the landscape connectivity can help in the recolonization of an area by the large carnivores [9]. Wolves can adapt to various habitats if the availability of prey animals is high enough and the anthropogenic pressure is limited [10]. Despite this, wolves can survive even at high human densities if there is effective wildlife management that protects them and ensures prey populations [11]. However, the spread of the species to human-dominated landscapes can increase the risk of human–carnivore conflicts [9,12].

The grey wolf has also been reappearing in the North Hungarian Mountains in Hungary since the 1990s [13,14]. Animals from neighbouring countries such as Slovakia, and even from the Czech Republic or Poland, could access Hungary due to their good ability to cover large distances during dispersal [15].

Recently, more and more game managers have reported wolf occurrences (tracks, prey remains) in their hunting units, showing a possible recolonization in this region. They have expressed frustration because wolves have decreased game populations and/or altered the predictability of their movements, leading to economic losses from hunting [13]. Wolves prey mainly on red deer in Central and Eastern Europe [16,17,18,19], and red deer is also an outstanding food item in Hungary [20]. The most favoured food source is presumably the red deer, mainly calves or weakened, vulnerable individuals [21]. The potential competition with hunters could be a reason to eliminate wolves in Europe [22,23], or at least more active wolf management is demanded by stakeholders like farmers and game managers [14]. To manage emerging conflicts while protecting the wolf population, more research on the impact of wolves on local prey populations should be carried out [22,24], and differentiated management measures should perhaps be implemented [5,14].

Large carnivores can influence the prey populations via direct mortality or indirect effects [25]; for example, frequent deer consumption was found in the Greater Yellowstone Ecosystem in the summer period [26]. Wolves can also have non-lethal effects on their prey species [27], for example, by altering the feeding habits, foraging patterns, vigilance, or habitat use of herbivores [28,29,30,31]. Lower energy income, energetic expenditure in defensive structures, decreasing mating success, more susceptibility to other predators, or emigration could be among the costs of defensive behaviour. These effects could be as strong on prey population dynamics as on the direct mortality [32]. Prey species exhibit a variety of predator avoidance behaviours, such as remaining vigilant while feeding, monitoring predators [33,34,35], altering their feeding activity [36], or adjusting the daily activity pattern to reduce predation risk [31,33,37,38]. Males were less sensitive to the presence of predators than, for example, calved females in both red deer and bison [39,40,41]. Female animals with calves are the most sensitive to predation risk, and it is no coincidence that this group responds most quickly. According to [40], females with calves show a strong response to predation risk because of the vulnerability of calves and increase their vigilance to protect their offspring.

Higher vigilance means an increase in the activity of the sympathetic nervous system, causing chronic stress [42,43] which can reduce individual fitness [44]. The higher glucocorticoid concentration during a prolonged time interval is related to the suppression of reproductive organs and the immune system, but it can also cause reduced growth, lower body condition, or starvation [45,46]. External factors affecting the condition of the animals may include the topography of the habitat or disturbance from different sources such as hiking, cycling, motorcycling, racing, skiing, logging, and drive hunts [47,48]. The greater defence against the predators may require a reduced time spent on foraging, which can result in lower body condition, higher mortality, and finally, small population size [28,49,50]. However, studies by [51] in Poland found that stress hormone levels were the opposite of what was expected. In the case of red deer, the level of faecal glucocorticoid metabolites was typically highest where no predators were present, and other factors such as the environment or anthropogenic influences were considered stronger. They also showed similar stress hormone levels in wolf areas as in those sites where predators did not occur. Similar results have been found for moose and wolves in Yellowstone, North America, where predators were not a chronic stressor for herbivores [52].

In this study, we first examined the distribution area of wolves in the North Hungarian Mountains in Hungary and then the influence of predator population on the condition and stress level of red deer individuals. The following three hypotheses were tested:

1. The distribution area of wolves in Hungary increased in the last two decades.
2. Red deer in wolf areas have higher levels of stress hormones compared to those in wolf-free areas due to their higher vigilance.
3. Red deer have decreased body condition in wolf areas compared to wolf-free areas due to the higher vigilance, shorter foraging time, or higher movement activity.

## 2. Materials and Methods

### 2.1. Study Area

The study was conducted in the North Hungarian Mountains in Hungary, which belong to the inner volcanic belt of the Northwestern Carpathians. This region lies along the border with Slovakia in a position of northeasterly direction from the line of Budapest. The total area is 13,000 km^2^. The highest peak of it is the Kékes (1014 m), located in the Mátra mountain range. The average temperature is 18–20 °C in July, while in January, it is −3–−5 °C. Precipitation is 700–800 mm/year; in winter, the landscape is covered with deep snow. Due to the abundant rainfall, there are extensive forests, dominated by oak species (*Quercus* spp.) and beech (*Fagus sylvatica*). Lots of streams and rivers can be found on volcanic rocks. Red deer, mouflon (*Ovis aries*), wild boar (*Sus scrofa*), and roe deer (*Capreolus capreolus*) are the main big game species in the area, but fallow deer (*Dama dama*) also occur in some places. The estimated population density and the harvested amount of these ungulates in 2021 in the study area can be seen in Table 1. We calculated the densities from the data of the National Game Management Database [53].

If the deer harvest density is compared to the German (0.23 deer/km^2^, [54]), Austrian (0.68 deer/km^2^, [55]), Slovenian (0.44 deer/km^2^, [56]), Polish (0.4 deer/km^2^, [57]), or the Czechian (0.42 deer/km^2^, [58]) data, it seems very high, while in Slovakia, for example, a similar value was found (1.01 deer/km^2^, [59]).

### 2.2. Mapping Wolf Distribution

In the last 20 years, three surveys have been carried out, asking all local game managers in the region whether grey wolves occur in their area. The result of the first such survey was summarised in 2006; then, the study was repeated in 2014, and finally, in 2021.

The data from these surveys were processed and compared using the geographic information software QGIS 3.20 to obtain a more accurate view of the re-establishment of the grey wolf. In order to investigate this, we used a map of Hungary based on the Universal Transverse Mercator (UTM) projection system. We only examined UTM cells of 10 × 10 km, with at least 25% of their area falling within the borders of our study area. Finally, they represented a total of 149 such cells, or 14,900 km^2^, as the study area. We examined the proportion of UTM cells where wolves were present based on the positive answers from the game management units reported regarding their area during the different survey periods. In the case of a positive answer, a threshold of 25% was set for overlap between the UTM cell and the game management unit for registering the wolf occurrence for that cell.

We assessed the frequency of wolf presence and its temporal variation in each UTM cell using a three-grade scale, with the categories ‘no occurrence’, ‘occasionally observed’, and ‘resident’. We looked at how the percentages of these classes changed over the different study periods and how the values varied within each UTM cell.

### 2.3. Measurement of Stress Hormone Level in the Red Deer Individuals

The great advantage of collecting and measuring faecal samples is that it is non-invasive and can be easily carried out by less experienced or even inexperienced persons. Moreover, as no capture is involved, any mishandling of individuals will not distort the results. In addition, it can also be used for samples from individual hunts, as the stress involved is not immediately reflected in the cortisol level of the faeces in the rectum, as a change in the faecal glucocorticoid metabolite (FGM) level of the carcass takes at least 6–24 h. There is another related advantage of this method. Compared to a blood measurement, which gives us a more instantaneous, current state, the FGM level in the faeces shows a slightly longer general period of about 1–2 days [60].

That is why, for stress hormone measurement, we collected faecal samples with sterile gloves from wolf-free areas and wolf areas in November and December 2021. Droppings from separate faeces groups on the ground were collected, ensuring as much independence as possible between sampled individuals. At least five pellets were gathered from each sampling patch. The samples were divided into four groups: (1) pellets from carcasses of hinds shot during individual hunts in wolf-free areas (n = 46); (2) pellets from carcasses of hinds shot during individual hunts in wolf areas (n = 49); (3) fresh red deer faeces collected from the ground in wolf-free areas (n = 31); and (4) fresh red deer faeces found on the ground in the wolf areas (n = 34). The samples were stored frozen at –18 °C. The samples obtained by hunting were only from females because [40] stated that females with young had a strong response to predation risk and increase their vigilance to defend their calves. That is why the largest stress response and therefore the most significant difference was expected in the hinds.

Stress hormone measurements were performed at the Endocrinology Laboratory of the University of Veterinary Medicine, Budapest, Hungary, using ELISA (Demeditec, Kiel, Germany), similarly to [48]. Characteristics of the analysis were intra-assay CV < 5% and inter-assay CV 8 and 6.3% (low [95 ng/mL] and high [225 ng/mL] control), respectively. For hormone extraction, 500 mg of the sample was placed in 4 mL of ethanol, vortexed for 15 min, and centrifuged at 3000× *g* for 30 min.

A Shapiro–Wilk normality test was used to analyse the normal distribution of the datasets. The hunted animals were only hinds, while the pellets collected from the ground could also originate from stags. That is why the samples derived from hunting were compared with each other separately with a Mann–Whitney U-test. The samples collected from the ground were tested with a parametric independent two-sample t-test after checking the similarity of the variances in the groups.

### 2.4. Measurement of Body Condition in the Red Deer Individuals

Wolves may more easily prey on roe deer and mouflon than red deer, but hunters’ experience showed that about as many red deer have been taken by wolves as mouflon, and far fewer roe deer, in our study region, although the roe deer population is significantly larger than the red deer population. We focused on red deer because local game managers were mostly interested in the impact of wolves on this species. That is why a kidney, kidney fat, and a long bone (tarsometatarsus) were collected from the carcasses of red deer hinds shot during individual hunts in the wolf and wolf-free areas to measure kidney fat index and bone marrow fat content (%). Two different indices were used together to increase the range of usefulness for the evaluation of the body condition of the red deer individuals [61]. The samples were obtained from November 2021 to January 2022. Samples were taken from 66 individuals from the wolf area and 67 individuals from the wolf-free area. The sample tissues were frozen at –18 °C until processing (maximum 1 month after hunting). The whole fat content around the kidney was removed together with the capsule of the organ. The kidney and the fat were weighed to the nearest tenth of a gram separately. The bone marrow was removed from the long bones and was measured to the nearest tenth of a gram (wet weight). The wet marrow was dried at 70 °C for 24 h until a constant weight was measured and weighed again (dry weight). The kidney fat index (KFI) was calculated according to the formula of [62] and bone marrow fat level (%) according to [61].

The body condition can vary with the age of the individuals [61], causing huge variance in the sample, which may mask the differences between the two area types. This is why the linear relationship between the estimated age of the hunted red deer hinds and their body condition indices was analysed. If there was no significant correlation between these characteristics of the animals, the kidney fat indices and the bone marrow fat levels were grouped, and then, these datasets were further analysed.

The Shapiro–Wilk test for kidney fat index and bone marrow fat content was used to verify the normal distribution of the data. The dataset of the kidney fat index followed a normal distribution in both areas, so we compared the two groups using a parametric independent two-sample *t*-test after checking the similarity of variances in the groups. Values of bone marrow fat level were not normally distributed, so the medians of the two groups were compared using a non-parametric Mann–Whitney U test.

The long-term stress situation caused by the predators could lower the body reserves of the prey animals. That is why the relationship between the stress hormone level and the body condition (KFI) of the hunted red deer hinds was analysed by Pearson correlation in the two areas separately. All statistical analyses were performed in SPSS 29.

## 3. Results

### 3.1. Wolf Distribution

**Figure 1:**
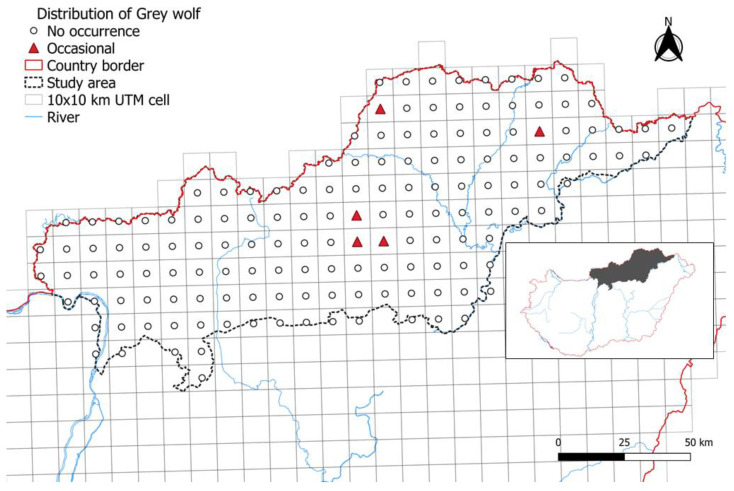
The presence of grey wolf in the study area in 2006.

The grey wolves occurred in 3.36% of the UTM cells surveyed in 2006. These cells represented 50,000 hectares, within which, wolves have always been occasionally observed (Figure 1).

**Figure 2:**
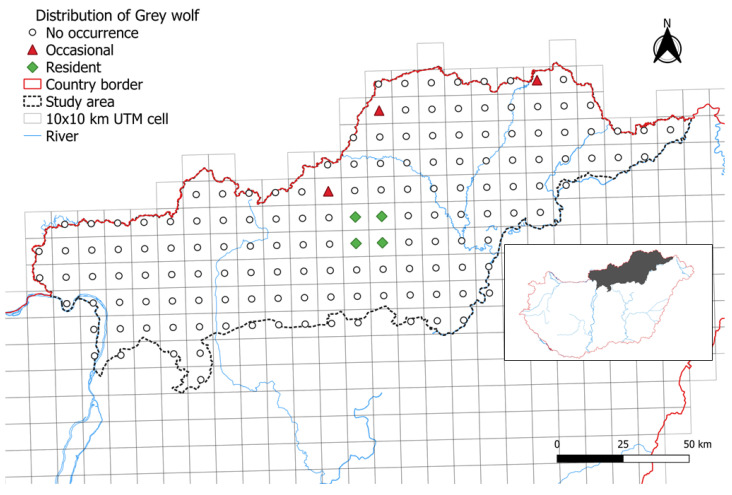
The presence of grey wolf in the study area in 2014.

The species was still reported to occur in only 4.7% of the surveyed UTM cells in 2014, totalling 70,000 hectares. However, at that time, the categorization of wolf presence changed to “resident” in slightly more than half (57.14%) of the positively reported cells (Figure 2).

**Figure 3:**
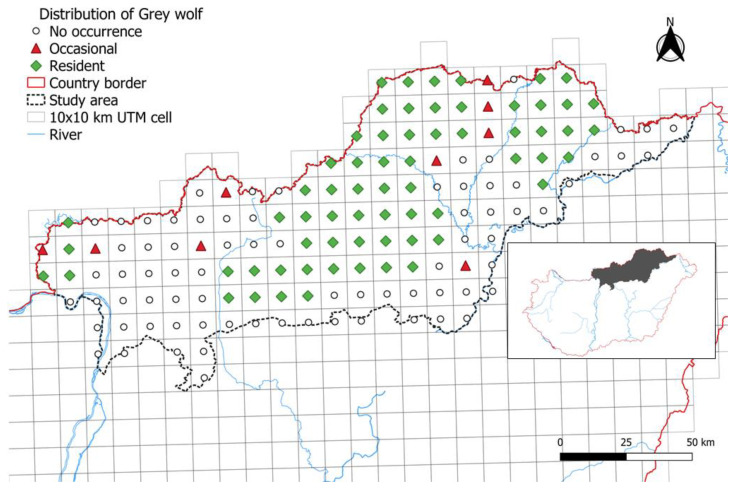
The presence of grey wolf in the study area in 2021.

But by 2021, game managers already confirmed the presence of the species in almost half of the area (49.66%), which represented 740,000 ha. Moreover, the overwhelming majority classified the wolves to be residents (87.84%) (Figure 3).

The comparison of the data from three consecutive questionnaire surveys are summarised in Table 2.

### 3.2. Stress Hormone Level

**Figure 4:**
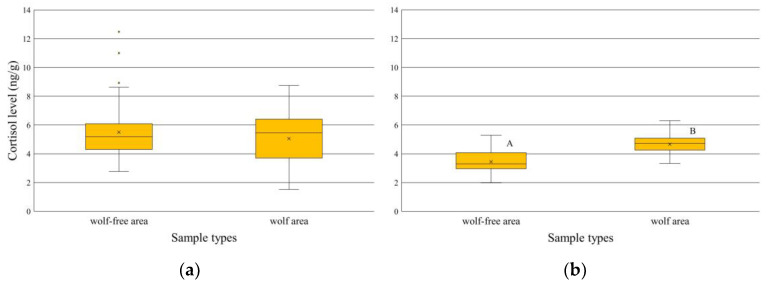
The cortisol level (ng/g) in the four sample types of red deer faeces. (**a**) The hunting samples collected from red deer hinds during individual hunts in wolf-free or wolf areas. (**b**) The ground samples were collected from the ground on transects in the two different areas. The different letters show significant differences (*p* < 0.001). The line in the boxes is the median, the x shows the mean, the box is the interquartile range around the median, the whiskers show 1.5 times the interquartile range from the box, and the points are the outliers or extreme values.

The comparison of samples from the hunts showed no difference in the cortisol level between the wolf-free and wolf area (U = 1118, n_1_ = 46, n_2_ = 49, *p* = 0.947) (Figure 4a). In contrast, the comparison of the stress hormone concentrations in the faeces from the ground confirmed a significant difference between the wolf and wolf-free sites (t = 7.074, df = 63, *p* < 0.001) (Figure 4b).

### 3.3. Body Condition

There was no significant relationship between the age of the animals and their body condition. The age of the individuals had no effect on the kidney fat index of the red deer hinds either in the wolf-free areas (F_1,64_ = 0.002, *p* = 0.964, R^2^ = 3 × 10^−5^) or in the wolf areas (F_1,56_ = 1.88, *p* = 0.176, R^2^ = 0.032). The bone marrow content was also not influenced by the age of the hinds in the two different areas (wolf-free sites: F_1,64_ = 0.297, *p* = 0.588, R^2^ = 0.005; wolf sites: F_1,56_ = 1.338, *p* = 0.252, R^2^ = 0.023). The body condition measures of the different age classes were grouped based on these results for the further analyses.

**Figure 5:**
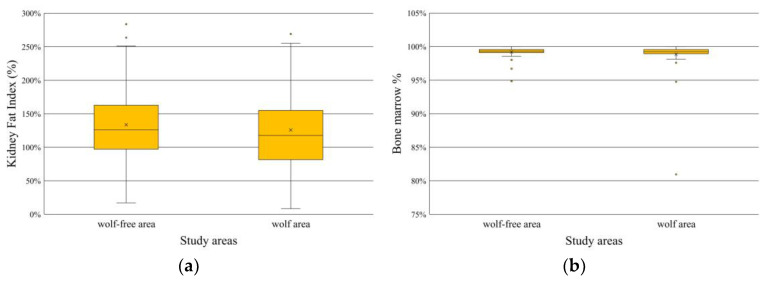
The body condition indices of the red deer hinds hunted in the two study areas. (**a**) The kidney fat indices of the red deer hinds. (**b**) The bone marrow fat contents (%) of the red deer hinds. The line in the box is the median, the x shows the mean, the box is the interquartile range around the median, the whiskers show 1.5 times the interquartile range from the box, and the points are the outliers or extreme values.

We did not reveal differences in the kidney fat indices of the red deer hinds between the two area types (t = 0.754; df = 129, *p* = 0.45) (Figure 5a). Similarly, the bone marrow fat content was not lower in the wolf area than in the wolf-free one (U = 1853, n_1_ = 67, n_2_ = 60, *p* = 0.448) (Figure 5b).

No significant correlation was found between the stress hormone level and the body condition (KFI) in the red deer hinds hunted in the wolf-free areas (r = 0.238, n = 45, *p* = 0.116) or in the wolf areas (r = −0.032, n = 45, *p* = 0.834).

## 4. Discussion

Our investigation on the occurrence of wolves in the North Hungarian Mountains confirmed the area expansion of this large carnivore during the last decade. This is in accordance with the statement of the report by the European Commission [63], that wolf distribution has increased rapidly since 2016 and large area gains have particularly been made in the Central European population.

Based on our results, it is not clear that the presence of wolves has a significant effect on the stress hormone (cortisol) levels of the red deer. Although cortisol levels were significantly lower in faecal samples collected from the ground in wolf-free areas than in other samples, we measured similar levels in hinds shot during individual hunts. One explanation could be that different timings of sample collection caused the discrepancy. A higher faecal glycorticoid level could be expected in winter because of the physiological mechanism of the body to mobilise the reserves to adapt to the higher energetic requirements of the cold winter conditions [64,65]. However, all the samples in our study were from the winter period, so there was no impact due to variability in the collection period.

The other explanation could be that the samples collected from the hunts and from the ground did not originate from the same location within wolf-free or wolf areas. Within those areas, the distribution of other important threatening factors besides wolves can vary influencing the results of stress hormone levels.

The studies by [51,52] support the idea that predators are not the main cause of high stress hormone levels in animals, but the human disturbance is the strongest factor. According to [47], the concentration of faecal cortisol level could be higher in areas where more stressors (in that study, more tourists) affect the animals. Similarly, the low wolf population density in our area could have a relatively lower impact than the impact of humans on the red deer individuals. Therefore, it is possible that the locations within the wolf-free area where droppings were collected from the ground may have been less disturbed by people (tourists, hunters) than all the other sampling sites.

Alternatively, perhaps there was an impact of wolves on the stress level of red deer, but an additional predator effect blurred the difference. Stray dogs may be less likely to be present in wolf areas since they can be preyed on by this large carnivore; therefore more dogs could occur in those locations of the wolf-free area from where samples arrived from huntings. Consequently, stress hormone levels in those areas increased to the level of wolf areas.

Another option could be that deer have become accustomed to the presence of wolves or have learned where the safer places are [66], so that this large predator no longer raises cortisol levels significantly compared to the effect of individual hunts. However, it should be noted that drive hunts have stronger effects on glucocorticoid levels in animals compared with individual hunts [67]. It should also be mentioned that the wolf population is still not large enough to have a spectacular impact on red deer. While we do not know exactly how many wolves may have been in the region, [15] suggest that there were at least 25 individuals in the mountains. However, according to [68], it could have been even 60–70 individuals in 2021/2022 in Hungary. Potentially, the consumption of livestock could have reduced the predation pressure on the wild prey species, and due to this, there was no clear difference in the stress hormone level of red deer between wolf and wolf-free areas. However, according to [69], wolves have shifted their diet in the past few decades by consuming more wild ungulates compared to previous records due to the large increase in wild prey species. These prey animals are supplemented by livestock or other anthropogenic food sources, where wild ungulates are scarce. But the population abundance of wild ungulates is much higher than that of the livestock in our study area, which is why livestock predation by wolves might not decrease the predation pressure on wild ungulates like red deer.

Deer stress hormone levels vary during the day, peaking at night as they are nocturnal mammals [70], which can affect the amount of glucocorticoid in the faeces. If the hinds were shot at different times, this could have also masked any differences that exist between the two areas.

Previous condition studies have all described the kidney fat index as a reliable condition trait [45,71,72]. Our study suggests that the condition of female red deer was not poorer in the wolf area, as shown by [50] in the Upper Gallatin study area. In both of our areas, the animals were in good condition (average KFI > 100%, [66]). In fact, the classification of bone marrow fat % showed that the hinds were in excellent condition (almost 100% bone marrow fat content) everywhere [73]. The good physical condition also confirms that the red deer were not under constant stress, because chronic stress results in the mobilisation of fat reserves [45]. This can also mean that the prey selectivity of wolves, meaning their behaviour of removing weak individuals, has no important role in this red deer population. It is probable that wolves tend to prey upon the weaker individuals. But recently, we only found red deer hinds in good body condition in the wolf areas. Perhaps this situation was the consequence of earlier selection by wolves, but it is also possible that wolves had no influence on this. However, if wolves consume individuals in good condition, this can increase the conflict between nature conservation and game management.

In addition, hinds should respond more strongly to the presence of large predators [40], i.e., stressful conditions may have a more severe effect on them. On the other hand, stress reduces the efficiency of digestion and storage, instead mobilising the body’s reserves [42,43,44,45], so one would expect that the fitness of females in the wolf areas to show this effect. In contrast, this was not evident; indeed, there was no significant difference in stress hormone levels between hunted female individuals.

## 5. Conclusions

In summary, through monitoring the impact of the landscape of fear, or at least of the wolves in this case, more precise predictions can be made about predator–prey relationships. Moreover, game managers or conservationists can better understand the regulatory effect of this repatriating large carnivore on ungulate populations. This can be the basis of common integrated management measures, leading to the best practises maintaining the favourable conservation status of the populations. As potential good examples, we can mention Slovenia, France [74], or the former Slovakian situation before 2021 [75]. In these countries, a quota system was established where wolves are strictly protected but problem individuals can be removed in a highly controlled way in some areas in specific conditions up to a predefined limit. Thus, animal breeders are receiving support and the possibility of reducing damage in a highly controlled way, in addition to preventive measures like guard dogs, electric fences, and compensations after wolf depredation. This could decrease the negative attitude towards wolves and help increase the toleration of their adverse impact.

Our partly contradictory results do not support a very intense recent impact of the wolf population on the body condition and stress level of red deer in Hungary. However, it is possible that this impact was more profoundly present during the intense stabilisation period of wolves after 2014. Further increases in large carnivore numbers can also sharpen those effects on the prey population. Moreover, due to the spread of African Swine Fever, the wild boar population had collapsed in this region, which could have led to the transfer of additional predation pressure on red deer. We can also suppose that wolves may have effects on the spatial distribution, movement pattern, activity, and vigilance behaviour of red deer. Additional studies are needed to reveal the diverse effects of wolves in this region to help wildlife managers and nature conservationists reduce conflicts connected to large carnivores.

## Figures and Tables

**Table 1:** Estimated population density and the harvest data of ungulate species in the study area in 2021.

Ungulate Species	Population Density (ind./km^2^)	Harvest Density (ind./km^2^)
Red deer	1.8	1.2
Fallow deer	0.2	0.1
Roe deer	3	1.1
Mouflon	0.3	0.1
Wild boar	0.5	1.3

**Table 2:** The number of UTM cells in the different occurrence categories in the three surveyed years and the rate of change (%) between the years.

Year	No Occurrence	Occasional	Resident
2006	144	5	0
2014	142	3	4
2021	75	9	65
2006→2014	–1.4%	–40%	-
2014→2021	–47.2%	+200%	+1525%

## Data Availability

The data presented in this study are available on request from the corresponding author due to the strictly protected status of wolves in Hungary.
